# Mitochondrial Oxidative Stress Regulates FOXP3+ T-Cell Activity and CD4-Mediated Inflammation in Older Adults with Frailty

**DOI:** 10.3390/ijms25116235

**Published:** 2024-06-05

**Authors:** Jappreet Singh Gill, Benu Bansal, Kai Guo, Fang Huang, Harpreet Singh, Junguk Hur, Nadeem Khan, Ramkumar Mathur

**Affiliations:** 1 Department of Geriatrics, School of Medicine and Health Sciences, University of North Dakota, Grand Forks, ND 58202, USA; jappreet.gill@und.edu (J.S.G.); benu.bansal@und.edu (B.B.); h.singh@und.edu (H.S.); 2 Department of Biomedical Engineering, School of Electrical Engineering and Computer Sciences, University of North Dakota, Grand Forks, ND 58292, USA; 3 Department of Biomedical Sciences, School of Medicine and Health Sciences, University of North Dakota, Grand Forks, ND 58202, USA; kai.guo@und.edu (K.G.); fang.huang@und.edu (F.H.); jung.hur@und.edu (J.H.); 4 Department of Neurology, University of Michigan, Ann Arbor, MI 48109, USA; 5 Department of Oral Biology, University of Florida, Gainsville, FL 32603, USA; nkhan2@dental.ufl.edu

**Keywords:** frailty, grip strength, regulatory T cell, inflammation, mitochondrial stress

## Abstract

In healthy older adults, the immune system generally preserves its response and contributes to a long, healthy lifespan. However, rapid deterioration in immune regulation can lead to chronic inflammation, termed inflammaging, which accelerates pathological aging and diminishes the quality of life in older adults with frailty. A significant limitation in current aging research is the predominant focus on comparisons between young and older populations, often overlooking the differences between healthy older adults and those experiencing pathological aging. Our study elucidates the intricate immunological dynamics of the CD4/Treg axis in frail older adults compared to comparable age-matched healthy older adults. By utilizing publicly available RNA sequencing and single-cell RNA sequencing (scRNAseq) data from peripheral blood mononuclear cells (PBMCs), we identified a specific Treg cell subset and transcriptional landscape contributing to the dysregulation of CD4^+^ T-cell responses. We explored the molecular mechanisms underpinning Treg dysfunction, revealing that Tregs from frail older adults exhibit reduced mitochondrial protein levels, impairing mitochondrial oxidative phosphorylation. This impairment is driven by the TNF/NF-kappa B pathway, leading to cumulative inflammation. Further, we gained a deeper understanding of the CD4/Treg axis by predicting the effects of gene perturbations on cellular signaling networks. Collectively, these findings highlight the age-related relationship between mitochondrial dysfunction in the CD4/Treg axis and its role in accelerating aging and frailty in older adults. Targeting Treg dysfunction offers a critical basis for developing tailored therapeutic strategies aimed at improving the quality of life in older adults.

## 1. Introduction

Aging induces notable alterations in immune functionality, such as thymic involution and a decline in T-cell receptor diversity [1,2]. However, the immune system in healthy older individuals preserves its homeostatic immune function, efficiently clearing pathogens and maintaining immunological balance [3,4]. Studies have shown the genetic, physiological, and environmental factors that contribute to frailty and pathological aging. Moreover, frailty is commonly associated with decreased immune surveillance by cells, including T cells and macrophages, resulting in persistent low-grade inflammation [1,5,6].

Notably, frailty often presents as a dysregulated immune response, exacerbating physical vulnerabilities such as diminished grip strength, an increased risk of falls, and a reduced healthy lifespan in older adults [7,8,9,10,11]. The dysregulation of CD4^+^ T cells has been shown to aggravate chronic proinflammatory cytokine production during aging [12,13,14,15]. Most studies have relied on traditional bulk RNA sequencing (RNAseq) or tissue and serum proteomics to explore frailty and chronic inflammation in older adults, comparing them to younger individuals [16,17]. These approaches often overlook the cellular and transcriptional heterogeneity of CD4^+^ T cells at the single-cell level, which is linked to the exacerbation of autoimmune inflammation in older adults with frailty. Identifying regulatory mechanisms that limit CD4^+^ T-cell responses in immune aging is crucial for improving beneficial outcomes in both healthy and frail older populations. FOXP3-expressing regulatory T cells (Tregs) are crucial for modulating CD4-driven inflammatory responses through various suppressive mechanisms, including the secretion of cytokines like IL-10 and interactions involving CTLA-4 [18,19,20,21,22]. However, age-related changes, such as metabolic shifts, thymic involution, and alterations in transcriptional programs, create a steady-state inflammatory environment that weakens the Treg suppressive ability [23,24]. This leads to immune dysregulation and increases chronic inflammation [23,24].

Moreover, elevated inflammatory conditions induce Treg plasticity, resulting in their conversion from suppressive cells to proinflammatory Th1 and Th17 helper cells, which results in dysregulated inflammatory responses [25]. However, some studies suggest that Tregs are resilient to such plasticity under inflammatory conditions [26,27,28]. Thus, extensive and unbiased single-cell research is required to understand Treg molecular changes, modulate CD4-mediated immune responses, and uncover novel treatment targets for inflammaging. Interestingly, recent studies have shown that mitochondria play a crucial role in controlling CD4 inflammation in aging by regulating cellular metabolism and maintaining cellular homeostasis [29,30,31]. As individuals age, mitochondrial function declines, leading to the increased production of reactive oxygen species (ROS) and oxidative damage. By analyzing publicly available bulk RNA-Seq and single-cell RNA-Seq data from PBMCs, we observed that a reduction in mitochondrial function with aging correlates with a substantial upregulation of CD4 inflammation and weakened cellular function in older adults with frailty compared to age-matched samples from healthy older adults. Our hypothesis suggests that the loss of mitochondrial proteins impairs FOXP3 Treg function and mediates CD4 inflammation associated with frailty. We further quantified frailty by identifying a frailty signature using a panel of genes associated with T-cell-specific inflammation, oxidative stress, and mitochondrial dysfunction. We also predicted the effects of gene perturbations on cellular signaling, revealing insights into the regulatory roles of transcription factors in T-cell differentiation and function. In addition, we observed that restoring mitochondrial activity with resveratrol and rapamycin reduced CD4 inflammation and reactive oxygen, suggesting a frailty intervention. Overall, this work highlights the age-related relationship between mitochondrial dysfunction in the CD4/Treg axis and immune dysregulation facilitating the acceleration of frailty. 

## 2. Results

### 2.1. Older Adults with Frailty Possess Elevated Levels of Peripheral Inflammation and a Pathological CD4 Landscape Compared to Healthy Older Adults

**Figure 1:**
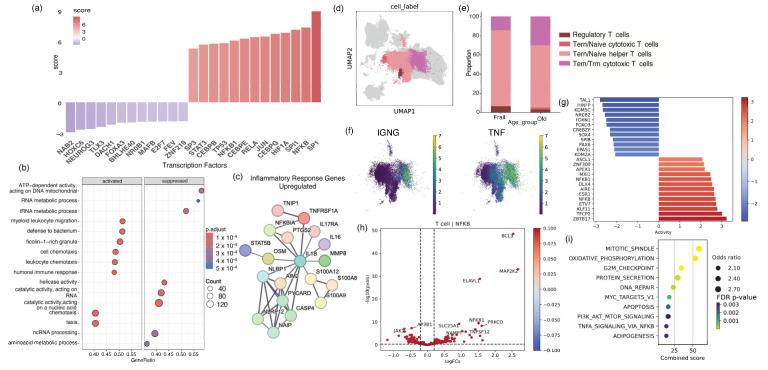
Older adults with frailty possess pathological CD4 landscapes compared to healthy older adults. (**a**) Transcription factor activity inference using a Univariate Linear Model (ULM) with PBMC RNA-Seq data from frail older vs. healthy older populations. (**b**) Pathway enrichment analysis using RNA-Seq DE gene sets from the frail population showing the top activated and suppressed pathways. (**c**) STRING network analysis showing upregulated genes involved in inflammatory response pathways in the frail group. (**d**) Uniform Manifold Approximation and Projection (UMAP) representing T-cell clusters from older adults with frailty and healthy older adults based on scRNAseq data. (**e**) A stacked bar plot indicating the distribution of T-cell subpopulations in scRNAseq data. (**f**) UMAP representing the expression of IFNγ and TNF in the T-cell subpopulation. (**g**) Transcription factor activity inference using a Univariate Linear Model (ULM) for PBMC scRNAseq T-cell clusters in frail older vs. healthy older populations. (**h**) Volcano plot depicting differentially expressed genes related to the NF-kB transcription factor in T cells. (**i**) Pathway analysis of T-cell-specific DE genes in frail older vs. healthy older populations based on scRNAseq data.

To examine the underlying molecular mechanisms linked to a compromised frail immune system, we conducted a comprehensive, unbiased analysis of publicly available bulk RNAseq (GSE206762) and cell-specific scRNAseq (GSE157007) peripheral blood mononuclear cell (PBMC) data from healthy older individuals and older adults with frailty [32,33]. For bulk RNAseq, we included 10 older adults with frailty with an average age of 81.0 (±5.0) and 10 healthy older adults with an average age of 78.8 (±2.6). Compared to healthy older adults, frail individuals showed substantial increases in proinflammatory markers, such as TNF, TNFR1, and IL6, and responsive transcriptional factors, including NF-kB, RELA, and STAT3. Moreover, there was a decrease in the expression of FOXOA3, NAE2, and BACH1 (Figure 1a). Moreover, compared to healthy older adults, individuals with frailty showed a decline in the mitochondrial metabolic pathway and elevated levels of oxidative stress and peripheral inflammation (Figure 1b). In a further comparison of older adults using a protein–protein interaction network, we found elevated gene levels of TNF-α, TNFR, Caspas4, and IL-1β proinflammatory cytokines and downstream signaling interlinked with accelerated cell death pathways in frail individuals (Figure 1c).

To better understand the cellular origins of systemic inflammation, we utilized scRNAseq data from the PBMCs of frail and healthy older adults. The publicly available data were collected from *n* = 5 sex-matched older adults with frailty with an average age of 88.0 (±5.8) years and *n* = 6 healthy older adults with an average age of 85.8 (±11.1) of similar demographics. By evaluating automatic cell annotation methods and analyzing cell markers, we successfully identified and measured the proportions of different cell types (Figure 1d,e). A comprehensive analysis of the cell-specific composition revealed a substantial decrease in Tem/Trm cytotoxic cells and an increase in regulatory T cells in frail individuals (Supplementary Figure S1). We further observed a considerable increase in the levels of the inflammatory cytokines TNF and IFNγ in specific clusters of cytotoxic CD4^+^ T cells (Figure 1f). In the CD4^+^ T cells of older adults with frailty, there were increased levels of Th1-type genes, such as those downstream of TNF, and a predominance of genes related to NF-kB, including BCL3 and MAP2K2, suggesting more inflammation in older adults with frailty compared to healthy older individuals. Interestingly, in older adults with frailty, the expression of the mediator JAK1, downstream of IL10, was markedly decreased (Figure 1h). It is worth mentioning that the expression of the transcription factors FOXO3, FOXN1, and SOX4 was much lower in the frail group than in healthy older individuals (Figure 1g). Additionally, the activity of the transcription factors ZBT17, TFCP2, and KLF11 had risen. FOXO3, FOXN1, and SOX4, which play critical roles in anti-inflammatory responses and are essential for regulatory T-cell stability, exhibited suppressive activity in older adults with frailty. Furthermore, we saw enrichment of pathways related to mitochondria, oxidative phosphorylation, and apoptosis (Figure 1i). Based on this analysis, higher levels of Th1-type cytokines and a decrease in cytokines that maintain anti-inflammatory responses, such as Treg maintenance and function, may induce pathogenic activity in older adults with frailty.

### 2.2. The Cellular and Functional Diversity of FOXP3+ Treg Cell Subtypes in Frail and Healthy Older Adults

**Figure 2:**
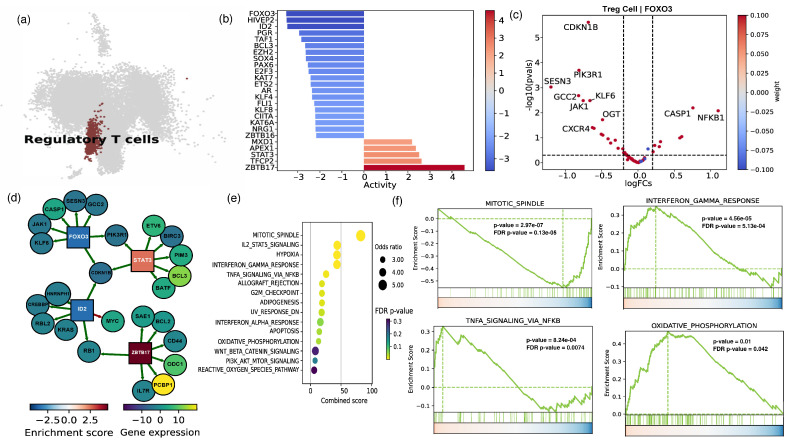
Cell–cell interactions associated with Treg responses in frail individuals. (**a**) UMAP highlighting the regulatory T-cell (Treg) population in frail older and healthy older samples. (**b**) Transcription factor activity inference in the frail population using a Univariate Linear Model (ULM) for the Treg subcluster. (**c**) A volcano plot depicting DE genes of the frail Treg population connected to the FOXO3 transcription factor. (**d**) A network plot visualizing the expression of the top 5 genes of selected enriched transcription factors in Tregs from frail older vs. healthy older adults. (**e**) A pathway analysis of the top DE genes in Treg cells from frail older vs. healthy older adults. (**f**) The GSEA plot depicts the enrichment scores for selected top pathways in Treg cells from frail older vs. healthy older adults.

Since Tregs play an essential role in controlling inflammation and preventing autoimmune reactions by suppressing the activation and functions of various immune cells, such as CD4^+^ T cells, a more in-depth analysis of scRNAseq data was performed to investigate the diversity of Treg cells and the mechanisms governing their response in frail versus healthy older adults [34,35,36]. We further created a UMAP plot to emphasize the Treg population in samples from older adults with frailty and healthy older adults (Figure 2a). Automatic cell annotation was used to identify the Treg cluster, which was then manually validated by the expression of FOXP3 and IL2RA (Supplementary Figure S2). We further conducted transcription factor activity inference in the frail population using a Univariate Linear Model (ULM) for Treg subclusters (Figure 2b). Surprisingly, in older adults with frailty, the activity and expression levels of transcriptional factors, such as FOXO3, ID2, and BCL3, were much lower than in healthy older adults (Figure 2b). This suggests that these transcriptional factors are necessary for Treg stability. We found that the high activation of ZBTB17, STAT3, and TFCP2 was related to an inflammatory response in Tregs (Figure 2b). Furthermore, the volcano plot shows the increased expression of inflammatory NFKB1 and CASP1 genes in the Treg population from frail older adults, which is potentially linked to the FOXO3 transcription factor (Figure 2c). Our findings indicate that decreased levels of the CDKN1B, PIK3R1, and CXCR4 genes are essential for maintaining anti-inflammatory activity and FOXO3 activation (Figure 2c). 

Furthermore, the top five genes regulated by the enriched transcription factors, including FOXO3, ID2, and ZBTB16 showed decreased expression in frail adults compared with healthy adults. The results revealed a predominance of inflammatory ZBT17 and STAT3 transcription factors (Figure 2d). The network analysis indicates that these transcription factors are primarily produced in T cells and potentially block the Treg-supporting pathway, regulating the genes involved in Treg modification in response to inflammatory circumstances. Next, we performed a pathway analysis of the top differential expressed (DE) genes in Treg cells from frail versus healthy older adults. It is worth mentioning that when Tregs from healthy aged individuals were compared to Tregs of older adults with frailty, the latter exhibited higher levels of inflammatory genes such as PCBP1, BCL3, and RAC1.

Finally, we used gene set enrichment analysis (GSEA) to determine the important pathways in Treg cells from frail vs. healthy older adults. Our data show that older adults with frailty have enhanced inflammatory TNF and IFNγ pathways relative to healthy individuals (Figure 2e,f). Moreover, our findings demonstrated that the frail group had a much higher level of gene enrichment linked to oxidative phosphorylation in Tregs than the elderly group. It is worth noting that oxidative stress is a crucial factor in the aging process [37,38]. Furthermore, we found a noteworthy reduction in the pathways connected with the mitotic spindle and their relationship with mitochondrial dysfunction and Treg stability, which are implicated in the development of proinflammatory Th1 responses. These findings suggest that specific transcriptional variables play an essential role in deciding the destiny of Tregs in frail individuals in the context of inflammation.

### 2.3. Assessing Cell–Cell Interactions Associated with Treg Response in Frail Older Adults

**Figure 3:**
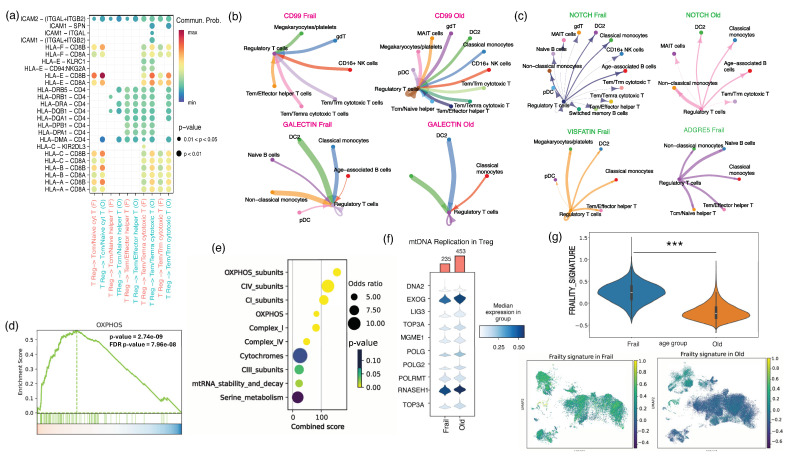
The cellular and functional diversity of FOXP3+ Treg cell subtypes in aging. (**a**) A dot plot indicating the Treg-specific communication probability of signaling molecules among T cells in frail older and healthy older populations. (**b**,**c**) A circle plot of the Treg-specific incoming and outgoing signaling strength of selected molecules in frail and healthy older adults. (**d**) A GSEA plot showing the enrichment score of a mitochondrial-specific pathway. (**e**) The top 10 enriched mitochondrial pathways in the T-cell population of frail individuals. (**f**) The median expression of genes involved in the mtDNA replication pathway in Tregs in the frail population. (**g**) A violin plot depicting expression differences in a gene signature for frailty and UMAP representing the expression of the frailty signature in all cell types. ***, *p* < 0.001.

Next, we employed CellChat to analyze ligand–receptor interactions in the scRNAseq data to determine potential cell–cell interactions between Tregs and other cell types. It is interesting to note that there is a distinct difference in the communication among T cells of frail vs. healthy older adults, as demonstrated by the dot plot for HLADQA1, HLADQA1, HLAC-CD8A, and HLAC-CD8B (Figure 3a). Also, we found potential differentially expressed pathways relevant for communication between cells (Supplementary Figure S3). Our studies found that Tregs in older adults with frailty possessed substantial incoming connections compared to T cells in healthy older adults. Subsequently, the interactions were assessed by calculating the average expression levels of receptors and ligands in the mentioned cell types. A comparison was made between ligands and receptors in cellular clusters of Tregs from frail and healthy older adults, as shown by a circle plot of the Treg-specific incoming and outgoing signaling strength of selected molecules (Figure 3b). 

Consequently, we employed network analysis and pattern recognition to statistically predict and assess intercellular communication networks of Tregs with other interacting cells. We found that inbound signals for CD99 and Galectin in Tregs increased in older adults with frailty, which is crucial for promoting inflammatory pathways. Surprisingly, we observed a substantial increase in outgoing signaling for VISFATIN and ADGRE in frail individuals, while no major interaction was revealed between VISFATIN and ADGRE signaling in healthy older individuals. Similarly, we found that frail adults exhibited more outgoing NOTCH signaling than healthy adults (Figure 3c). According to previous studies, VISFATIN and ADGRE increase the control of inflammation and death in cells [39,40,41]. Studies have shown a correlation between the decline in genes found in mitochondria and the subsequent increase in oxidative stress and inflammation [42,43,44]. Evidence suggests that elevated OXPHOS can contribute to oxidative stress and activate inflammatory pathways [45,46]. 

Our analysis revealed an intriguing upregulation of the mitochondrial OXPHOS signaling pathway in the Treg cells of older adults with frailty compared to healthy older adults (Figure 3d). In our study, we analyzed the top 10 enriched mitoCarta mitochondrial pathways in the Treg population of older adults with frailty compared to healthy older adults (Figure 3e). Mitochondrial dynamics can influence the maintenance of the mitotic spindle’s stability and organization. This suggests that any changes in mitochondrial dynamics due to aging can impact its overall function. In addition, our analysis uncovered an uptick in OXPHOS and the activation of complex I and complex IV pathways in Tregs. Research has shown a strong correlation between the number of mitochondria and the preservation of mitochondrial function. Furthermore, we observed a decline in genes associated with mitochondrial replication in the Tregs of frail individuals (Figure 3f). Based on the above analysis and findings, we created a gene signature for frailty. Upon comparison, we found a substantial, statistically significant upregulation of the gene signature in frail individuals (Figure 3g). Additionally, we generated a UMAP plot to visualize the expression of the frailty signature across various cell types (Figure 3g). Our analysis revealed a major increase in frailty signature genes among T cells of frail compared to healthy older adults.

### 2.4. Restoring Mitochondrial Oxidative Stress Alleviates Inflammation in In Vitro CD4^+^ T Cells 

**Figure 4:**
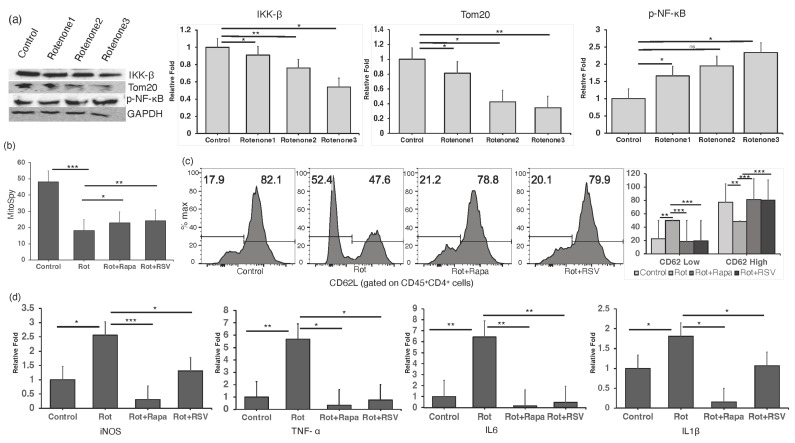
The restoration of mitochondrial oxidative stress alleviates inflammation in vitro in CD4^+^ T cells. (**a**) CD4^+^ T cells were treated with various doses of rotenone (1 nM, 10 nM, 100 nM) for 24 h. Cell lysates were analyzed by Western blotting with the indicated antibodies. (**b**) CD4^+^ T cells were preincubated for 16 h with rapamycin (10 µM) and resveratrol (10 µM). Cells were then treated with rotenone (10 nM) for 24 h, stained with Mitospy, and analyzed by flow cytometry (FACS). Data were analyzed using FlowJo software (version 10.4.0) and are represented in a bar diagram. (**c**) CD4^+^ T cells were gated on CD45^+^CD4^+^CD62L^+^ and analyzed by FACS, with statistical values plotted. (**d**) CD4^+^ T cells were preincubated for 16 h with rapamycin (10 µM) and resveratrol (10 µM). Cells were then treated with rotenone (10 nM) for 2 h. The transcript levels of TNF-α, IL-6, IL-1β, and iNOS were detected by qPCR analysis. *, *p* < 0.05; **, *p* < 0.01; ***, *p* < 0.001; ns, *p* > 0.05.

We investigated the impact of mitochondrial dysfunction on cellular health and oxidative stress, which leads to an inflammatory response in CD4^+^ T cells. Purified CD4^+^ T cells were treated with varying doses of rotenone, a well-established inhibitor of mitochondrial complex I known to induce inflammatory responses and upregulate reactive oxygen species (ROS) production. Our study revealed that rotenone treatment led to a dose-dependent inflammatory response, as evidenced by the degradation of IκBβ and an increase in NFκB levels, shown through Western blot analysis using anti-IκBβ and anti-NFκB antibodies. The mitochondrial content was assessed with an anti-TOM20 antibody, and blots were normalized to GAPDH, confirming an enhanced inflammatory signal in CD4^+^ T cells (Figure 4a). To further assess mitochondrial health, we used Mitospy dyes, which are cationic, lipophilic fluorescent probes that accumulate in the mitochondria of living cells in proportion to the mitochondrial membrane potential. Flow cytometry of CD45^+^CD4^+^ cells stained with Mitospy revealed a significant reduction in mitochondrial membrane potential and cellular health upon rotenone treatment (Figure 4b). We further evaluated inflammatory gene expression in CD4^+^ T cells by qPCR analysis and observed the upregulation of TNF-α, IL-6, IL-1β, and inducible nitric oxide synthase (iNOS) (Figure 4d).

Previous studies have indicated that resveratrol, a polyphenolic antioxidant, significantly reduces oxidative stress [47,48]. We validated our findings by restoring mitochondrial function through the treatment of CD4^+^ T cells with resveratrol and rapamycin, an mTOR inhibitor with anti-inflammatory properties, followed by rotenone (10 nM) treatment. qPCR analysis was conducted after 2 h, and Western blot and FACS analyses after 24 h. Our results demonstrated that rotenone treatment led to the increased activation of CD4^+^ T cells (CD4^+^CD62L^low^). However, resveratrol and rapamycin treatment significantly reduced CD4^+^ T-cell activation and restored the reduced activation observed in CD4^+^CD62L^high^ cells (Figure 4c). Additionally, the combined rotenone and rapamycin treatment reduced the expression of TNF-α, IL-6, IL-1β, and iNOS induced by rotenone (Figure 4d).

Our findings suggest that rotenone-induced mitochondrial dysfunction promotes oxidative stress and inflammatory responses in CD4^+^ T cells. Overall, these results support the hypothesis that reduced mitochondrial function compromises cellular health and elevates inflammatory responses in CD4^+^ T cells. Restoring mitochondrial health alleviates CD4^+^ T-cell dysfunction and CD4-driven inflammation.

### 2.5. In Silico Knockout Modulating the Perturbation Score for the Treg Gene Signature

**Figure 5:**
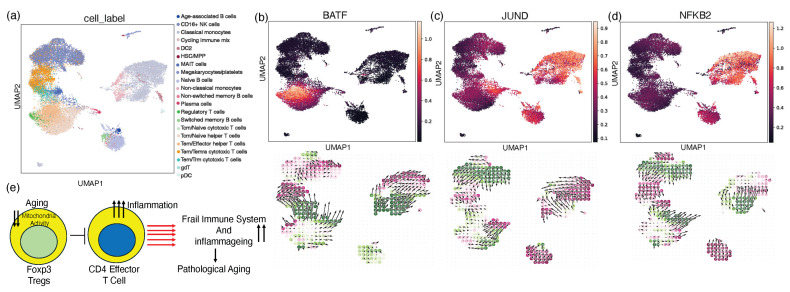
In silico knockout modulating the perturbation score for the Treg gene signature. (**a**) The UMAP representation of cell clusters used for gene perturbation analysis. (**b**–**d**) Using computer modeling and simulation via a perturbation-based in silico KO method, we predicted the impact of gene perturbation (BATF, JUND, and NFKB2) on cellular pathways, activities, and phenotypes in Tregs in older adults with frailty and healthy older adults. The arrows represent the direction of cell differentiation. (**e**) A working model for research demonstrating that defective Tregs in frail individuals activate CD4 T cells, with mitochondrial protein loss being a significant determinant of illness outcomes.

To investigate the potential regulatory roles of the transcription factors BATF and JUND in T-cell differentiation and function, we leveraged the in silico gene perturbation capabilities of the CellOracle computational platform [49]. CellOracle is a genome-scale modeling framework that integrates single-cell transcriptomics data with curated gene regulatory networks (GRNs) to infer context-specific gene regulatory interactions. We simulated the knockout of BATF and JUND individually and assessed the perturbation scores for key T-cell subsets, including regulatory T cells (Figure 5a). Our in silico analyses revealed that the knockout of the BATF gene resulted in a negative perturbation score for the Treg gene signature, suggesting that BATF acts as a positive regulator of Treg development or function (Figure 5b). Our findings are consistent with previous research showing that BATF is needed for the induction and maintenance of the Treg transcriptional program [50,51]. Tregs serve a vital role in reducing inflammation and maintaining immunological homeostasis; thus, perturbations that affect Treg stability and plasticity may lead to autoimmune and inflammatory diseases [52]. In contrast, the knockout of JUND led to a positive perturbation score for Tregs, indicating that JUND may play an inhibitory role in Treg biology (Figure 5c). This finding aligns with reports that JUND can antagonize the activity of FOXP3, the master transcriptional regulator of Tregs [53,54]. The opposing effects of BATF and JUND knockouts on the Treg state imply a complex interplay between these two transcription factors in controlling T-cell differentiation and the inflammatory response. Further, we investigated the effect of NFKB2, which is known to regulate the inflammatory response, and found decreased perturbation, implying the downregulation of Treg differentiation (Figure 5d). These computational predictions provide a foundation for further experimental validation of the roles of BATF and JUND in regulating Treg plasticity and the Treg-mediated control of inflammation. Our research proposes a working model that illustrates how defective Tregs in frail individuals contribute to the activation of CD4^+^ T cells (Figure 5e). A critical factor in this process is the loss of mitochondrial proteins, which plays a decisive role in influencing disease outcomes. This model underscores the complex interplay between immune dysfunction and mitochondrial impairment in shaping the health trajectory of older adults with frailty.

## 3. Discussion

The mounting incidence of immune dysfunction among the growing population of older adults with frailty presents a critical health concern. With the global aging population expected to reach 2.1 billion by 2050, it is imperative to concentrate on aging immunology to develop strategies that leverage the aging immune system and meet the evolving healthcare needs of this demographic [55]. Frailty in older adults is often accompanied by immune dysregulation and increased inflammation, with mitochondrial dysfunction playing a pivotal role in these processes. In this study, we leveraged publicly available bulk and single-cell datasets to expand our understanding of the role of mitochondrial dysfunction and immune dysregulation in older adults with frailty, specifically focusing on CD4^+^ T cells [32,33,56]. Our findings offer novel insights and therapeutic possibilities that expand on our existing understanding of immune regulation in frailty.

Studies have shown the critical role of mitochondrial energy metabolism in Foxp3+ T-regulatory (Treg) cells, identifying key metabolic regulators such as Pgc1a and Sirt3, which are crucial for Treg function and allograft survival [56,57,58]. They demonstrated that enhancing mitochondrial respiration through Hdac9 deletion improves Treg function, suggesting new therapeutic strategies for immune modulation [56]. Another study identified a distinct expression pattern of circular RNAs (circRNAs) between frail and robust elderly individuals, highlighting their potential as biomarkers for frailty [32]. Innovations in single-cell RNA sequencing have enabled the comprehensive profiling of immune cells across various conditions, revealing an age-dependent accumulation of transcriptome heterogeneity in immune cells and identifying specific transcription factors and distinct cellular trajectories associated with aging [59,60]. Furthermore, a frailty-specific monocyte subset characterized by the high expression of the long noncoding RNAs NEAT1 and MALAT1 provides new insights into immune senescence [33]. These findings underscore the dynamic changes in immune cell composition and functionality with age, highlighting the intricate variability in the immune response in frailty.

Our research expands this understanding by specifically examining how mitochondrial oxidative stress affects FOXP3+ T-cell activity and CD4-mediated inflammation in older adults with frailty. This focus provides new insights into the cellular mechanisms underlying immune dysregulation and frailty, highlighting the significant impact of mitochondrial dysfunction on these processes. Additionally, our study introduces resveratrol as a potential therapeutic agent to restore mitochondrial function and stabilize Treg cells, offering a novel approach to mitigating the effects of frailty. This therapeutic angle complements and extends the findings of existing research by proposing a specific intervention to address mitochondrial dysfunction, thereby opening new avenues for treatment. Our research further extends our current understanding of this condition by developing a comprehensive frailty signature using a panel of genes associated with T-cell-specific inflammation, oxidative stress, and mitochondrial dysfunction. This signature provides a quantitative measure of frailty-associated molecular features, enhancing the diagnostic and prognostic capabilities in the context of aging and immune health.

Our results highlight a substantial increase in CD4 inflammation and a reduction in Treg activity in older adults with frailty, pointing to a complex interplay of proinflammatory mechanisms within the immune system. Conventional aging research has often limited its focus to comparing the immune systems of older adults with those of younger individuals, frequently overlooking the molecular changes in immune cells among healthy older adults during natural aging [61,62,63,64]. Addressing this gap, our study underscores a potential link between diminished grip strength and elevated inflammation in frail adults compared to their healthier counterparts. Through comprehensive analyses of unbiased publicly available bulk RNAseq and cell-specific scRNAseq data of peripheral blood mononuclear cells (PBMCs) from age-matched healthy older adults, we have observed heightened levels of peripheral inflammation and a disrupted CD4 landscape in frail individuals. These subjects demonstrated significant increases in proinflammatory markers, such as TNF, TNFR1, and IL6 and transcription factors like NF-kB, RELA, and STAT3. Notably, frail individuals exhibited declines in mitochondrial metabolic pathways, accompanied by increases in oxidative stress. Further exploration of scRNAseq data from the PBMCs of sex-matched frail and healthy older adults revealed enhanced levels of the inflammatory cytokines TNF and IFNγ, signaling the dominance of NF-kB-related pathways. These transcriptional alterations suggest an increased proinflammatory state with a concurrent decrease in the anti-inflammatory Treg population in frail individuals compared to healthy older adults.

Treg plasticity, observed under inflammatory conditions enriched with IFN and TNF, remains a critical area of research. Despite the known impact of inflammation on Treg conversion in the peripheral blood of frail individuals, the functional implications and regulatory mechanisms of Tregs in suppressing such responses need further exploration [25,52]. The dynamic nature of Tregs, responding to a milieu of inflammatory signals, transcriptional regulation, and epigenetic modifications, underscores the complexity of immune regulation in aging [65,66]. Our study has identified the downregulation of key transcription factors, like FOXO3, ID2, and BCL3, and the upregulation of ZBTB17, STAT3, and TFCP2, which are pivotal in controlling Treg behavior under inflammatory conditions. This imbalance contributes to the regulatory challenges in maintaining Treg functionality and highlights the necessity for targeted interventions to restore immune tolerance in autoimmune and inflammatory disorders. Moreover, our analysis using CellChat to explore ligand–receptor interactions across the scRNA-seq dataset has illuminated an intricate network of cell–cell interactions, particularly highlighting a substantial increase in VISFATIN/ADGRE signaling in frail individuals, which is known for its proinflammatory properties. We observed that reductions in mitochondrial proteins are intrinsically linked to increased oxidative stress and cellular senescence, exacerbating age-related pathologies. Changes in mitochondrial dynamics potentially affect the stability and organization of the mitotic spindle, thereby implicating mitochondrial function in the maintenance of cellular integrity.

Interestingly, the reduction in mitochondrial proteins is a critical intrinsic factor involved in increased oxidative stress, cellular senescence, and age-related pathologies [67,68]. Alterations in mitochondrial function and abundance trigger an increased production of reactive oxygen species (ROS) and lead to oxidative damage to cellular components [69,70]. CD4^+^ T cells, which rely on mitochondrial metabolism, are particularly vulnerable to these changes. Age-related changes in mitochondrial dynamics can affect the stability and organization of the mitotic spindle, potentially impacting its maintenance. We noted an elevation in OXPHOS and the activation of complex I and IV pathways in Tregs, suggesting a strong correlation between mitochondrial function and the abundance of mitochondria. Further investigation into how inflammation triggers TNFR1 activation in CD4^+^ T cells revealed that prolonged exposure to TNF-α activates inflammatory pathways, which can be mitigated by the antioxidant properties of resveratrol. This finding supports the potential therapeutic use of antioxidants like resveratrol to reduce oxidative stress and inflammation.

Lastly, we employed computational modeling, and the perturbation-based in silico knockout approach utilized in our study provides a novel and powerful tool for understanding the complex interactions between gene expression and immune function in aging populations. Our prediction that the induction of inflammatory genes like JUND and NF-kB negatively impacts the Treg phenotype and that their knockout alleviates Treg suppression highlights potential therapeutic targets for addressing immune dysregulation in the elderly. These findings are particularly significant given the role of Tregs in maintaining immune homeostasis and preventing chronic inflammation, which is a common feature of aging and frailty [71,72]. By focusing on specific inflammatory pathways, our research opens new avenues for the development of targeted therapies that can enhance Treg function and improve overall immune health in elderly individuals. Enhancing mitochondrial function has the potential to mitigate the physiological deterioration associated with aging, thereby improving quality of life. Overall, our research highlights the crucial role of targeted, gene-specific interventions in managing frailty and extending the health span of aging populations. This work paves the way for innovative therapies that can effectively address the unique challenges faced by the elderly.

## 4. Materials and Methods

### 4.1. Cell Culture

CD4^+^ T cells were isolated from the spleens of wild-type C57BL/6 mice. Spleens were mechanically disrupted against a 40 µm mesh filter to produce a single-cell suspension. Red blood cells were lysed using ACK lysis buffer, and the remaining cells were stained with a CD4-PE-conjugated antibody followed by PE-conjugated microbeads, in accordance with the manufacturer’s instructions. The stained cells underwent magnetic separation to enrich for CD4^+^ T cells. The purified CD4^+^ T cells were then cultured in Dulbecco’s Modified Eagle Medium (DMEM) supplemented with 10% fetal bovine serum and 1% penicillin/streptomycin. Cultures were maintained at 37 °C in a humidified atmosphere containing 5% CO_2_. For experimental treatments, CD4^+^ T cells were exposed to varying concentrations of rotenone (1 nM, 10 nM, and 100 nM) for 2 h for quantitative PCR (qPCR) analysis and for 24 h for Western blot analysis. Additionally, cells were pre-treated with rapamycin and resveratrol for 16 h prior to rotenone exposure (10 nM) for subsequent analysis by qPCR after 2 h and fluorescence-activated cell sorting (FACS) after 24 h.

### 4.2. Flow Cytometry Analysis

A single-cell suspension was prepared from the mouse spleen. To purify CD4^+^ T cells, we incubated splenocytes with an anti-CD4 antibody using the following protocol: Add 1 µL of anti-CD4-PE antibody per 10^6^ cells to the cell suspension at the recommended dilution. Incubate the cells with the antibody on ice for 20–30 min. Add anti-PE microbeads (20 µL of beads per 10^7^ cells). Place the cell suspension in a magnetic field and collect the labeled CD4^+^ T cells. Wash the collected CD4^+^ T cells and count and assess the CD4^+^ T cells using flow cytometry. In order to evaluate CD4^+^ T cells, we utilized single-cell suspensions and conducted cell surface staining in 50 µL of FACS buffer (1% BSA, 2 mM EDTA, and 0.1% sodium azide in PBS, pH 7.4) (5–10 × 10^5^ cells/tube). Following this, we incubated the cells with antibodies for a duration of 30 min at 37 °C. Then, 10^6^ cells/50 mL were incubated with anti-CD45 PercpCy5.5 (Cat#103132, Clone#30F11, BioLegend, San Diego, CA, USA), antiCD4 FITC (Cat#100408, Clone#GK3.5, BioLegend), and anti-CD62L PeCy7 (Cat#104418, Clone# MEL/4, BioLegend) on ice for 30 min. After staining, the cells were carefully washed twice with 500 µL of ice-cold FACS buffer to remove any bound antibodies. The cells were then promptly analyzed using a BD FACSymphony Flow Cytometer (BD Biosciences, Franklin Lakes, NJ, USA). MitoSpy staining (Cat# 424801, BioLegend) was performed following the manufacturer’s instructions. Cells were suspended in MitoSpy in 300–400 µL of warm 1X PBS and incubated at 37 °C for 30 min. After the cells were washed twice with 1X PBS, they were analyzed using a BD FACSymphony Flow Cytometer on both stained and unstained samples (BD Biosciences, Franklin Lakes, NJ, USA).

### 4.3. RNA Isolation and qPCR

We extracted total RNA from mouse tissues or single cells by mincing them with scissors and adding 500 µL of TRIzol reagent (Ambion, cat# 15596018, Life Technologies, Carlsbad, CA, USA). The Fisher Scientific Sonic Dismembrator 100 (American Laboratory Trading, East Lyme, CT, USA) was used to homogenize tissue solutions. After removing the top layer, RNA was precipitated in isopropanol and incubated at 55 °C for 10 min before resuspending in 50 µL of RNase-free water (Promega, Madison, WI, USA). A Bio-Rad Smart-Spec plus spectrophotometer (Bio-Rad Laboratories, Hercules, CA, USA) was used to measure RNA concentrations. Next, the High-Capacity cDNA Reverse Transcription Kit (Cat#: 4368814, Applied Biosystem, Thermo Fisher Scientific, Waltham, MA, USA) was used to generate cDNA from 0.5–1 µg of total RNA in 25 µL. The RT-PCR procedure ran for 5 min. at 25 °C, 30 min. at 42 °C, and 30 min. at 95 °C. Water was used to dilute cDNA templates two to three times. Two microliters of diluted templates and a Bio-Rad 96-well PCR plate (Cat#: MLL9601, Bio-Rad, Hercules, CA, USA) were used for qPCR. Each sample was tested in triplicate using a 2X SYBR Green PCR mix (Cat#: B21202, Bimake TE Huissen, The Netherlands). All genes were amplified twice. The CT value was used to determine RNA abundance fold changes after normalization to Gapdh. The Bio-Rad Aria Mx Real-Time PCR instrument was used to perform qPCR experiments.

### 4.4. Western Blot

Cultured and treated purified CD4^+^ T cells were lysed in RIPA lysis buffer containing a 1% protease inhibitor cocktail. The protein concentration was determined using the Bio-Rad protein assay reagent according to the manufacturer’s instructions and our previously published protocol. Proteins were separated by SDS-PAGE and electroblotted onto an Immobilon-P transfer membrane (cat# IPVH00010). The immunoblot was blocked for 1 h at room temperature with 5% nonfat dry milk in TBST (25 mM Tris-HCl, pH 7.4, 1.5 M NaCl, 0.05% Tween-20), followed by overnight incubation with primary antibodies at 4 °C. The next day, the membranes were washed five times at six-minute intervals and incubated for one hour with an HRP-conjugated secondary antibody (A16172, Invitrogen, Carlsbad, CA, USA) (1:1000) in 3% nonfat dry milk in TBST. The blots were then washed five additional times with TBST and developed with enhanced chemiluminescence. The following primary antibodies were bought from Cell Signaling Technologies (Cell Signaling Technology, Danvers, MA, USA): IkBb (Cat#8943T), Tom20 (Cat#424046), pNF-kB (Cat#96874T), and GAPDH (D16H11) XP^®^ Rabbit mAb #5174.

### 4.5. Bulk RNA-Seq Data Analysis

The publicly available bulk RNA-Seq data were retrieved from the National Center for Biotechnology (NCBI) Gene Expression Omnibus (GEO) database (accession GSE206762) [32]. Gene count matrices for all samples, including samples from 10 frail and 10 healthy older adults, were extracted for further analysis. The data were loaded into R, and the standard DESeq2 protocol was used to find differentially expressed genes [73]. Following the DESeq2 results, transcription factor activity inference analysis was conducted using the decoupleR Univariate Linear Model (ULM) with the CollecTRI gene regulatory network [74,75]. The pathway analysis utilized the Cluster Profiler package in R to elucidate the biological pathways associated with the identified gene sets [76]. The list of upregulated differentially expressed genes was inputted into the STRING web interface, which employs a comprehensive collection of experimentally validated and computationally predicted protein interactions from various sources and was used to construct the protein–protein interaction (PPI) network [77]. The interaction networks were then generated based on a confidence score threshold, where interactions above a predefined confidence level of 0.7 were considered reliable for further analysis. The PPI network was further skimmed by keeping only the genes involved in the inflammatory response pathway.

### 4.6. Single-Cell RNA-Seq Data Processing

Single-cell data were retrieved from the NCBI GEO database (accession GSE157007) [33]. The data were preprocessed using the scVI (single-cell variational inference) framework in Python (3.11.5) [78]. Subsequently, the scVI model was applied to the preprocessed data to perform normalization, the imputation of dropouts, and batch correction, ensuring the harmonization of gene expression profiles across different samples and experimental conditions. One sample (F013) was removed from further analysis, as it seemed like an outlier after integration. Further, samples from frail (*n* = 5) and healthy (*n* = 6) older adults were retained for subsequent analysis. Additionally, to mitigate the influence of potential doublets or multiples, a crucial preprocessing step involved the removal of putative doublet cells using the scVI doublet detection module, which leverages a probabilistic approach to identify and filter out cells exhibiting characteristics consistent with doublet formation. This rigorous preprocessing pipeline ensured the generation of high-quality scRNA-seq data, enabling the downstream analysis of cellular heterogeneity, the identification of cell types, and the exploration of transcriptional dynamics at a single-cell resolution.

### 4.7. Single-Cell Data Cell-Type Annotation

The automatic cell clustering of single-cell RNA sequencing (scRNA-seq) data was performed using the majority voting parameter of CellTypist, which utilizes a machine learning approach to classify cell types based on their transcriptomic profiles [79]. CellTypist leverages a reference atlas of known cell types to assign labels to individual cells within the dataset, facilitating the identification of distinct cell populations and their associated gene expression signatures. The PBMC-specific cell atlas from CellTypist was used as a reference dataset. Following automatic clustering, the assigned cell types were confirmed and validated manually using PanglaoDB-curated cell markers [80]. This manual validation process involved examining the expression patterns of marker genes specific to known cell types within each cluster, thereby ensuring the accuracy and reliability of the automated cell-type assignments. By combining the computational power of CellTypist with manual curation based on established marker genes, this integrated approach enabled the unbiased, robust, and comprehensive characterization of cellular heterogeneity within the scRNA-seq dataset, providing insights into the composition and functional diversity of the cell populations under study.

### 4.8. Single-Cell Data Differential Gene Expression Analysis, Transcription Factor Inference, and Pathway Analysis

Differential gene expression analysis at the pseudo-bulk level, the inference of transcription factor activity, and pathway analysis were performed using the decoupleR package (version 1.5.0) in Python [74]. First, pseudo-bulk samples were generated from the scRNA-seq data to facilitate differential expression analysis across experimental conditions. The decoupleR package was employed to identify genes differentially expressed between conditions utilizing DESeq2 algorithm. Subsequently, transcription factor activity inference was conducted using the decoupleR Univariate Linear Model (ULM) approach with the CollecTF gene regulatory network database to infer the activity levels of transcription factors across samples. Additionally, a pathway analysis was performed using the Over-Representation Analysis (ORA) method with the MSigDB hallmark database, allowing for the identification of biological pathways enriched with differentially expressed genes and transcription factor targets. Further, to characterize mitochondrial function, a pathway analysis focused on mitochondrial pathways was conducted using the MitoCarta database [81]. MitoCarta is a comprehensive collection of genes encoding mitochondrial proteins, curated from various sources, including experimental studies and computational predictions.

### 4.9. Cell–Cell Communication Analysis

Cell–cell communication in scRNAseq data was analyzed using the CellChat (version 1.6.1) package in R (version 4.3.1) [82]. The scRNAseq data underwent quality control and normalization to ensure reliable and comparable gene expression measurements across cells. Subsequently, the CellChat package was applied to the preprocessed data to infer cell–cell communication networks. The ligand–receptor interaction database was set to all, including secreted signaling, ECM–receptor, and cell–cell contact. The package leveraged curated databases and the literature to identify known ligand–receptor interactions, enabling the inference of potential communication events between different cell types within the scRNAseq dataset. By integrating these interactions into comprehensive communication networks, CellChat facilitated the characterization of complex regulatory mechanisms governing cell behavior and tissue homeostasis.

### 4.10. Gene Signature and Perturbation Analysis

A frailty signature was developed to capture the molecular underpinnings of frailty using a selected panel of genes derived based on our discussed findings, including those associated with T-cell-specific inflammation, oxidative stress, and mitochondrial dysfunction. The selected genes (*SSU72*, *GRK6*, *PCBP1*, *BCL3*, *RAC1*, *ODC1*, *SUN2*, *CMPK1*, *COTL1*, *NFKB1*, *ELAVL1*, and *NDUFAF3*) were identified based on their differential expression patterns in scRNAseq data analysis. A signature score was calculated using the sc.tl.score_genes function implemented in the Scanpy toolkit (version 1.9.6) to quantify the frailty signature across individual cells. This function computes a summary score reflecting the aggregate expression levels of the selected genes within each cell, thereby providing a quantitative measure of frailty-associated molecular features. In silico knockout experiments were conducted using the CellOracle framework to predict the effects of gene perturbations on cellular signaling networks [49]. CellOracle utilizes machine learning algorithms and cell–cell communication models to simulate the impact of gene knockouts on intercellular signaling pathways. This approach predicts how specific gene perturbations influence signaling dynamics and cellular responses within the biological system under study. It quantitatively compares the directionality and size of vectors between the perturbation simulation and natural differentiation using the inner product and defines the score as the perturbation score. A negative perturbation score (PS) (annotated in red) means that the TF perturbation would block differentiation, while a positive PS (annotated in green) means that the TF perturbation would promote differentiation.

## 5. Conclusions

This study elucidates the immune dynamics between healthy and older adults with frailty and pathological aging. Most published studies have overlooked the distinction between healthy older adults and older adults with frailty of comparable ages. Here, we conducted a comprehensive analysis of publicly available unbiased human PBMC bulk RNAseq and scRNAseq data to establish that Treg dysfunction in frail older adults results in an unrestricted CD4 landscape and elevated levels of peripheral inflammation compared to healthy older adults. Our findings offer insights into the cellular and functional diversity, as well as cell–cell interactions associated with FOXP3+ Treg cell subtypes, in frail and healthy older adults. In parallel, our mouse model experiments involved isolating CD4 T cells and inducing mitochondrial dysfunction using rotenone, a mitochondrial complex I inhibitor. This experimental reduction in mitochondrial function resulted in heightened inflammation, comparable to the observations in human samples. Notably, this research highlights the potential of targeted therapeutic strategies, such as resveratrol intervention, to restore mitochondrial function and Treg stability, thereby mitigating the effects of frailty. Overall, this study advances our understanding of the biological underpinnings of frailty and underscores the necessity of developing interventions that effectively regulate the CD4/Treg axis. These interventions could offer potential therapeutic targets to mitigate inflammaging, improve lifespan, and promote healthy living in older adults with frailty.

## Data Availability

The publicly available datasets used in this study are accessible at GEO (https://www.ncbi.nlm.nih.gov/geo (accessed 14 March 2024)) as GSE206762 and GSE157007 [32,33].

## Supplementary Materials

The supporting information can be downloaded at: https://www.mdpi.com/article/10.3390/ijms25116235/s1.
