# Supplementary material for: Mitochondrial Oxidative Stress Regulates FOXP3+ T-Cell Activity and CD4-Mediated Inflammation in Older Adults with Frailty
Source: Int J Mol Sci. 2024 Jun 5;25(11):6235. doi: 10.3390/ijms25116235 (PMC11173216; doi:10.3390/ijms25116235)
Supplement: Supplementary file 1 [file ijms-25-06235-s001.zip › ijms-3003242-supplementary.pdf]

Supplementary Figure S1. (**a**) Volcano plot depicting bulk RNA-Seq top differentially expressed genes in frail old vs. healthy old population. (**b**) UMAP distribution of single-cell data cell types. (**c**) A stacked barplot shows the composition of all the cells in the dataset. (**d**) Boxplot comparing cell type composition in frail old and healthy old populations. (**e**) Volcano plot depicting differentially expressed genes connected with the ID2 transcription factor. (**f**) A bulk RNA-Seq UMAP plot depicts the sample distribution.

Supplementary Figure S2. (**a-d**) UMAP and dot plot of marker genes used to validate T cell subtypes.

Supplementary Figure S3. (**a**) Barplot shows the total number of interactions for cell-cell communication among all cell types in frail old and healthy old populations. (**b**) Barplot depicts the strength of interactions for cell-cell communication among all cell types in frail old and healthy old populations. (**c**) Signaling changes of Regulatory T cells in frail old vs. healthy old populations with color representing the age group and shape representing the type of signaling. (**d**) Inverted stacked Barplot of overall information flow of signaling molecules between older adults with frailty (red) and healthy old (blue) populations. (**e**) Scatter plot visualization of the dominant senders and the receiver for incoming and outgoing signaling in frail old and healthy old. The dot color represents the cell type, and the dot size represents the count. (**f**) Chord diagram representing the number of interactions between Tem/Effector helper T cells and Regulatory T cells among older adults with frailty and healthy old.
