# Supplementary figures and images for: Mitochondrial Oxidative Stress Regulates FOXP3+ T-Cell Activity and CD4-Mediated Inflammation in Older Adults with Frailty
Source: Int J Mol Sci. 2024 Jun 5;25(11):6235. doi: 10.3390/ijms25116235 (PMC11173216; doi:10.3390/ijms25116235)

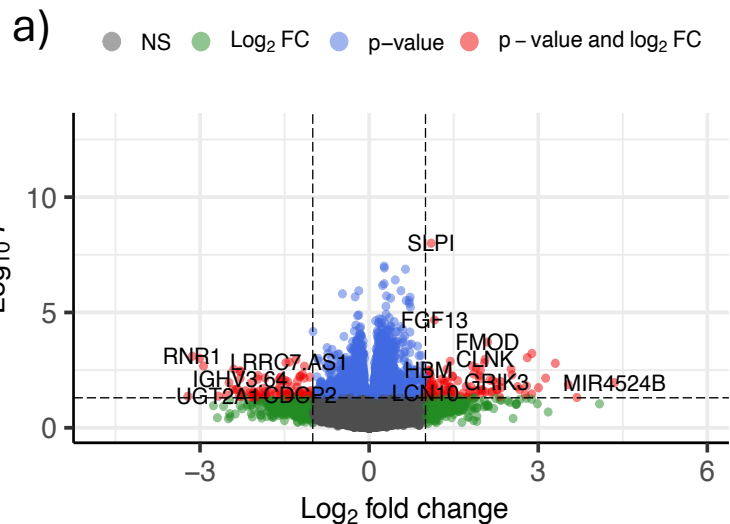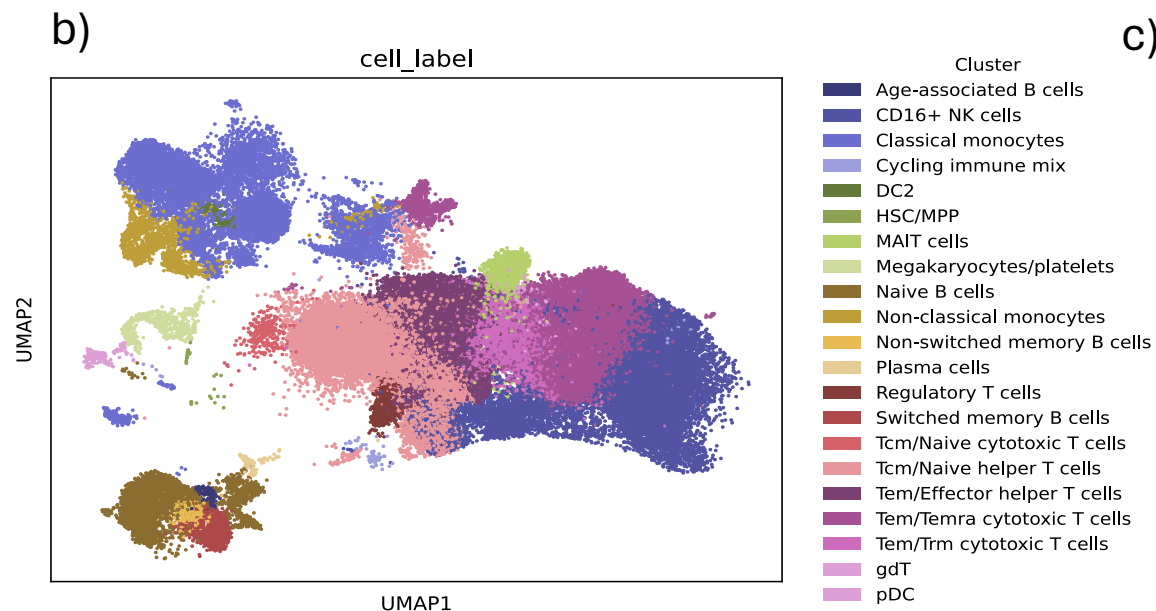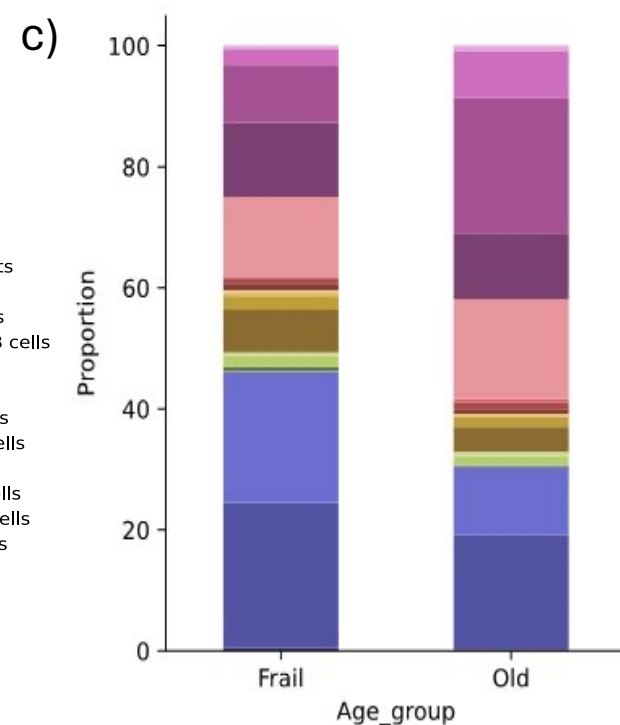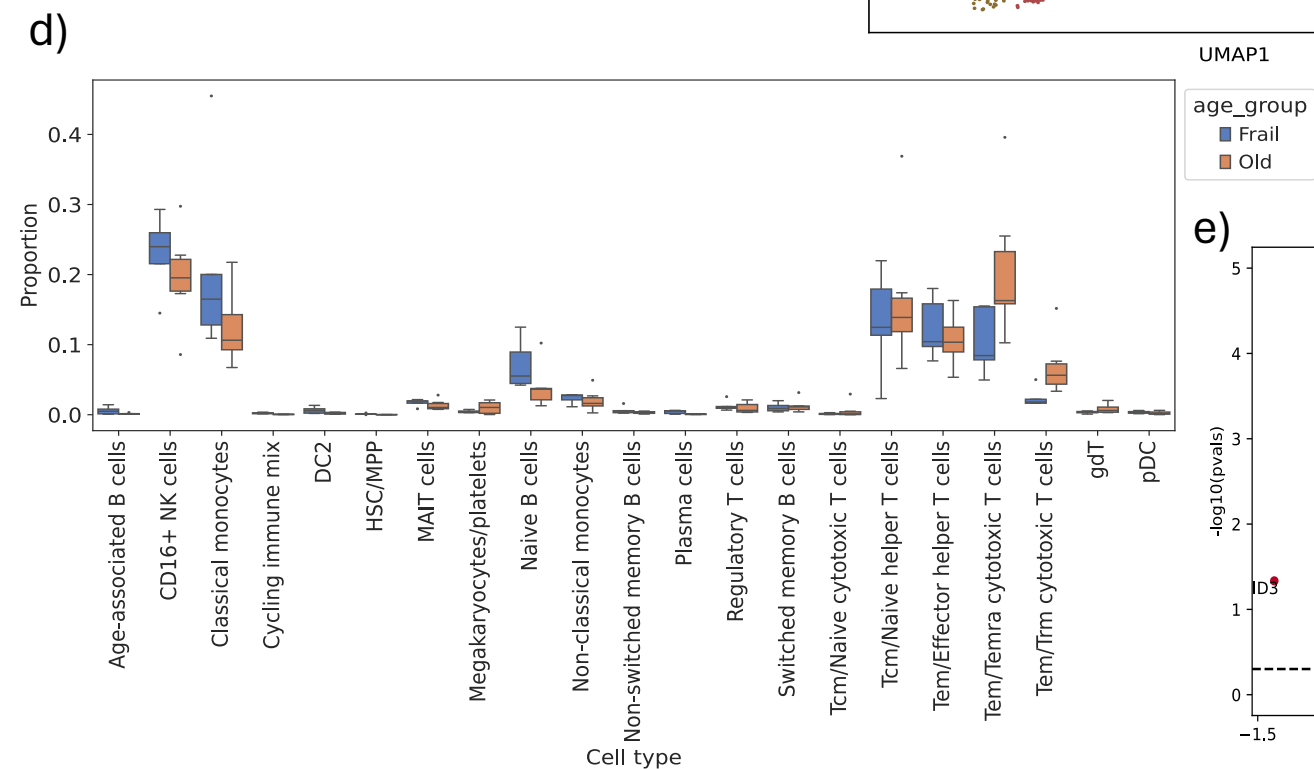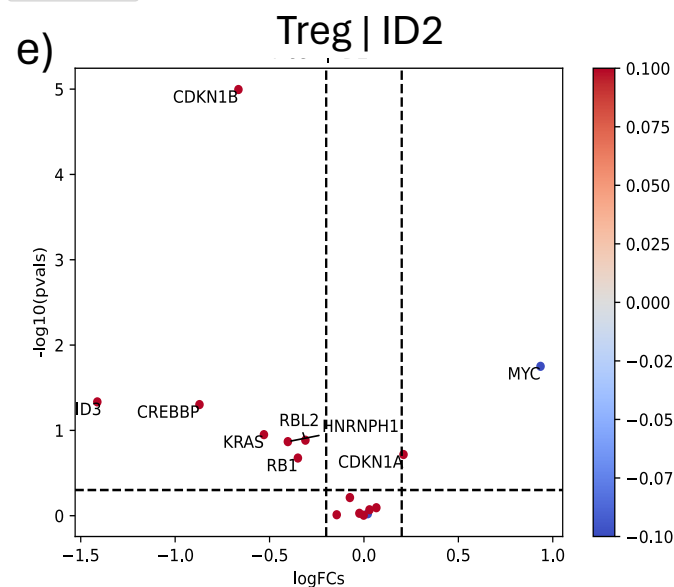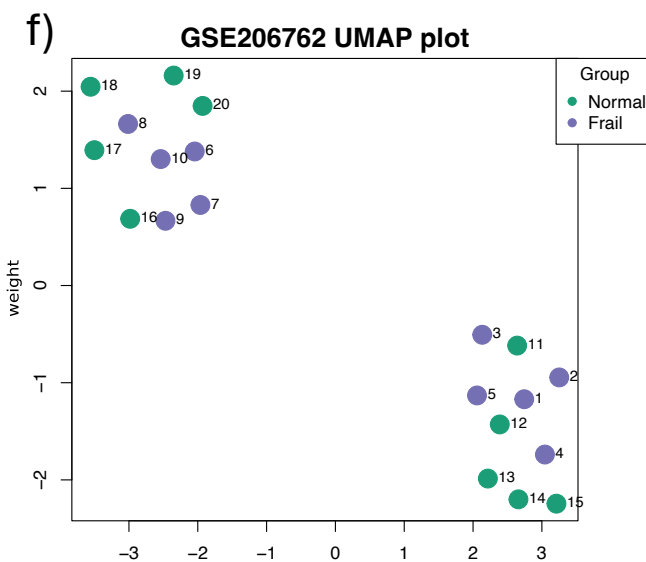

Supplement: Supplementary file 1 [file ijms-25-06235-s001.zip › Supplementary FigureS1.pdf]

# T cell subtype Annotation

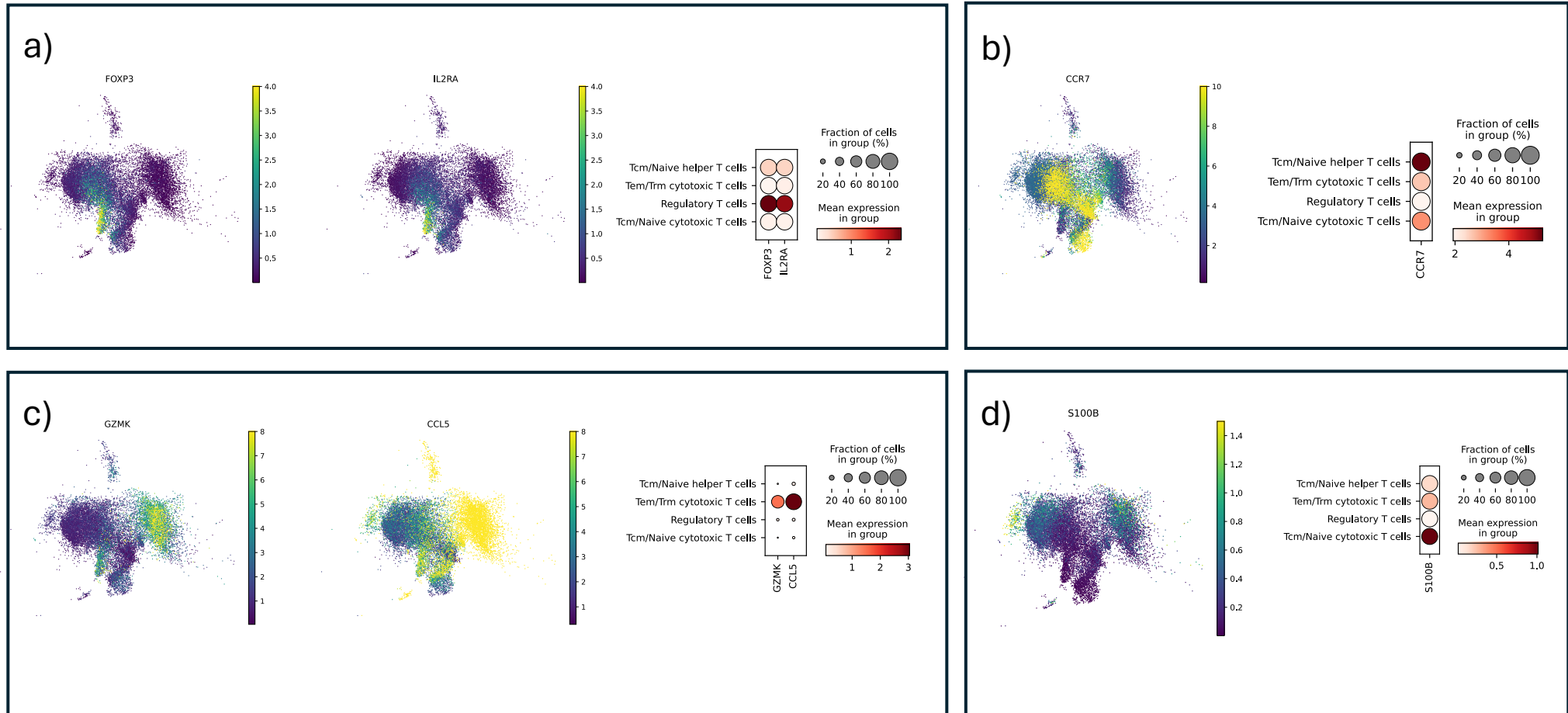

Supplement: Supplementary file 1 [file ijms-25-06235-s001.zip › Supplementary FigureS2.pdf]

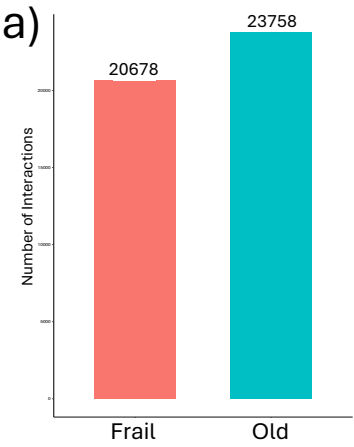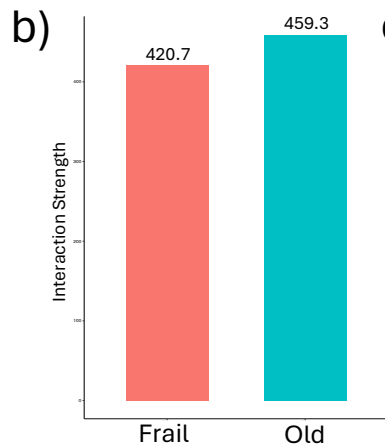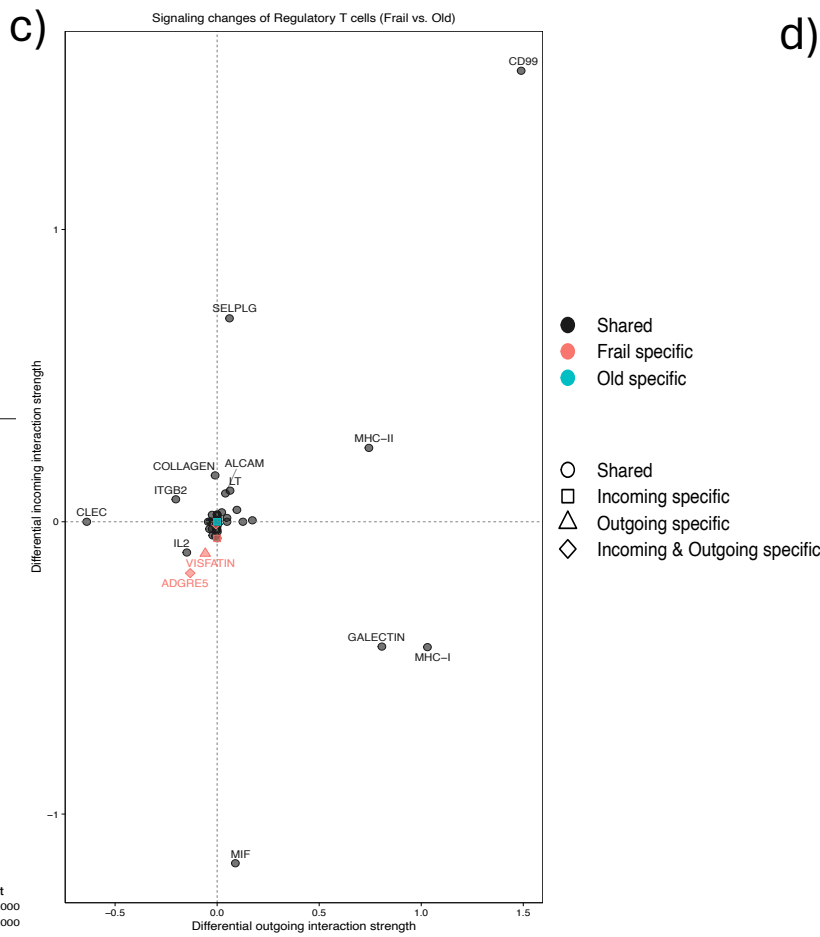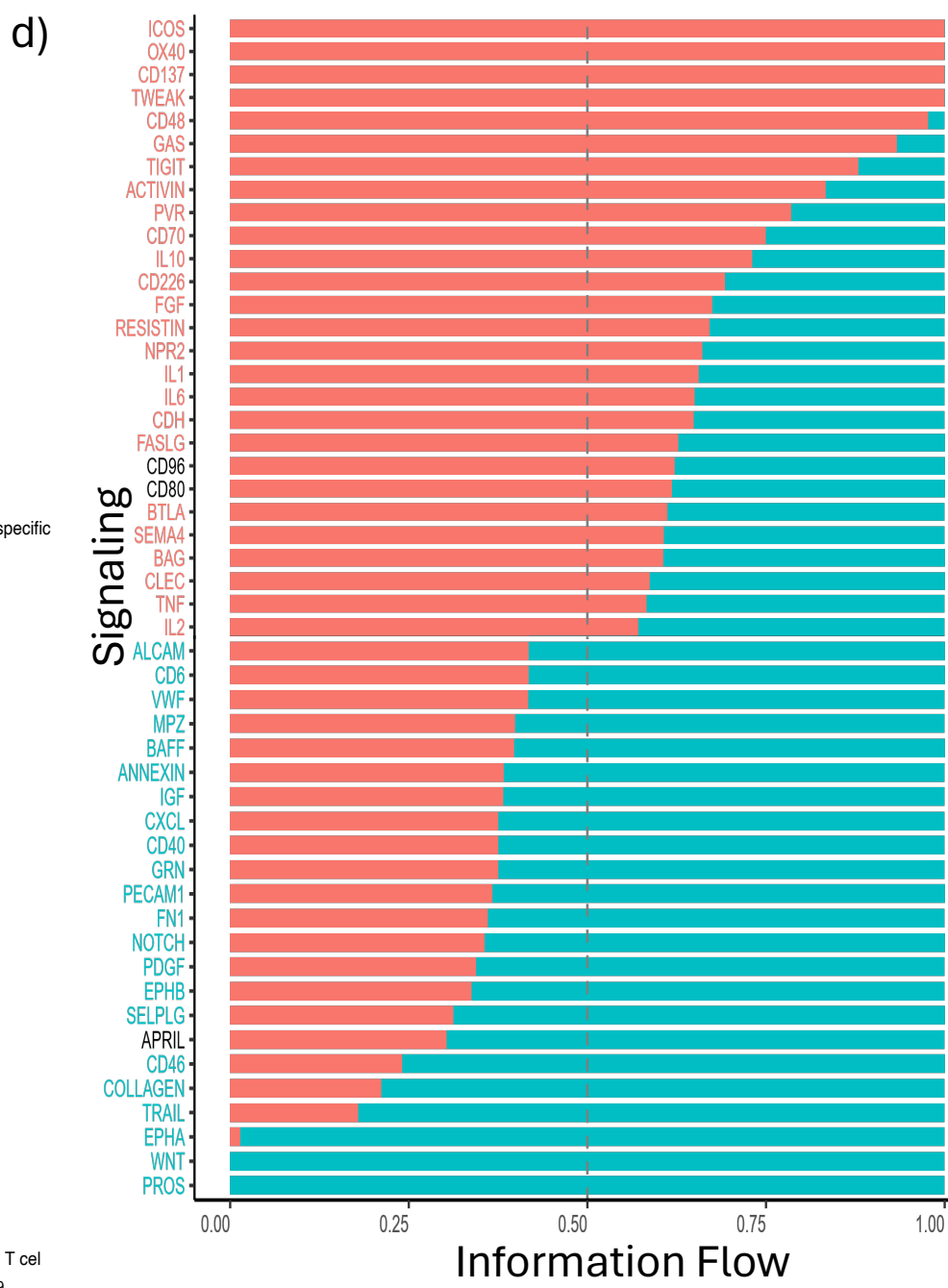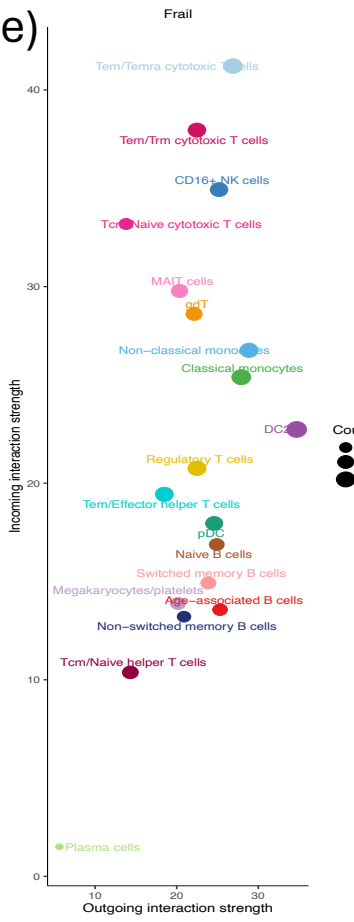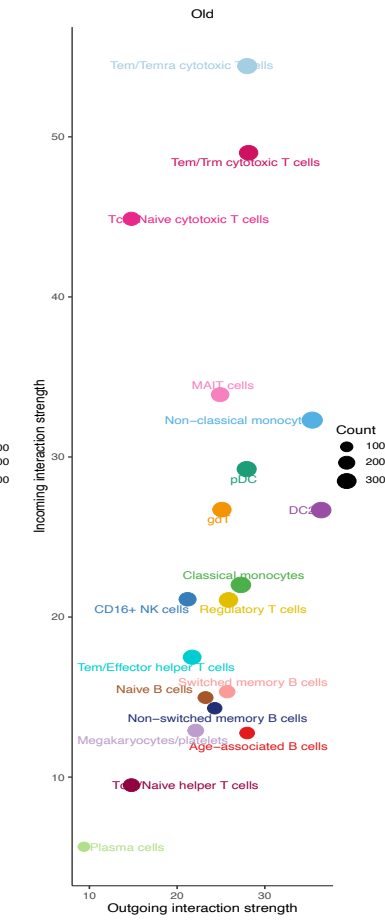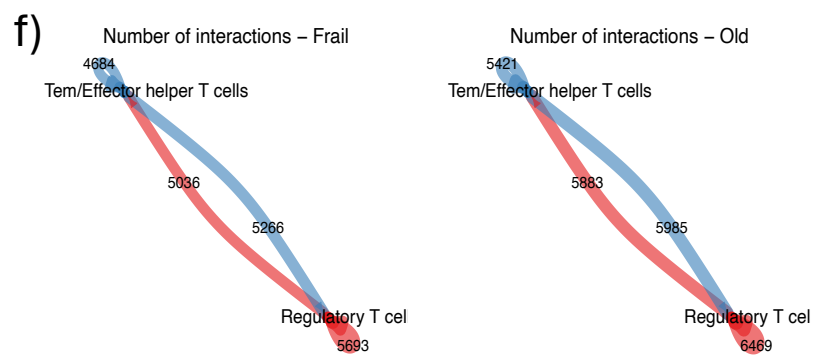

Supplement: Supplementary file 1 [file ijms-25-06235-s001.zip › Supplementary FigureS3.pdf]
